# Cultural Distance and Social Needs: The Dynamic Adjustment Mechanisms of Social Support Among Newly Arrived Students in Hong Kong

**DOI:** 10.3390/bs15091231

**Published:** 2025-09-10

**Authors:** Shiyi Zhang, Qi Wu, Xuhua Chen

**Affiliations:** 1 Department of Sociology, Harbin Institute of Technology, Harbin 150001, China; 1155219987@link.cuhk.edu.hk (S.Z.); 2021110931@stu.hit.edu.cn (Q.W.); 2 Department of Sociology, The Chinese University of Hong Kong, Hong Kong SAR, China

**Keywords:** newly arrived students (NAS), social needs, social networks, homophily, friendship

## Abstract

Based on questionnaire data and in-depth interviews with newly arrived students (NAS) from mainland China, this study investigates the construction of their social networks and the mechanisms through which they access social support in the context of migration. Drawing on Berry’s acculturation theory, Bronfenbrenner’s ecological systems theory, and Bourdieu’s concept of social capital, this study provides a theoretically grounded analysis of how NAS balance cultural distance and social needs. The findings reveal that NAS do not form social connections uniformly; rather, they strategically allocate social resources according to the degree of homophily and the strength of social ties. Specifically, weak ties with mainland peers—characterized by high cultural homophily—primarily offer emotional support; strong ties with local Hong Kong peers—marked by low homophily but high interaction frequency—mainly serve instrumental needs such as academic assistance and daily companionship; while strong ties with Hong Kong peers of mainland background combine both emotional and instrumental support, functioning as a core relational bridge in the NAS’s adaptation process. These three types of relationships form a complementary structure within NAS’s social networks. Reliability and validity tests further confirmed that four items (social satisfaction, peer attitude, sense of belonging, integration/adaptation) provide a coherent measure of social integration. The study suggests that NAS’s social practices are not merely about “integration” or “alienation,” but rather represent a dynamic strategy of balancing relational costs, cultural distance, and practical needs in the operation of social capital and characterised by dynamic negotiation and contextual adjustment.

## 1. Introduction

In recent years, the number of Newly Arrived Students (NAS) in Hong Kong has steadily increased, drawing growing attention from both educational and social research communities. However, despite this growing attention, existing studies have mainly focused on academic performance, family background, and identity, while less attention has been paid to the structure and functions of NAS’s peer networks. According to the Hong Kong Education Bureau, in 2023, the number of cross-border students exceeded 32,000, with NAS accounting for over 40% at the secondary school level. This trend signals not only educational concerns but also highlights the broader challenges of social adaptation. NAS refer to students who have migrated from Mainland China to Hong Kong within the past three years. They often face structural challenges during the adaptation process, including language barriers, cultural dissonance, and the difficulty of rebuilding social networks. These factors collectively impact their ability to integrate socially and emotionally into the new environment. Although previous research has examined NAS’s educational trajectories, family influences, and identity formation, less attention has been paid to their social behaviours and the structure of their social networks. Particularly lacking is a systematic analysis of how NAS navigate between cultural distance and social needs to form relationships that fulfil both emotional and instrumental support.

To address this gap, the present study examines NAS’s strategies for building social networks and accessing support in contexts shaped by cultural heterogeneity and varying interaction intensities. Specifically, the study asks: How do NAS construct and mobilize peer networks to meet emotional and instrumental needs under cultural distance? The study is theoretically grounded in Berry’s (1990) acculturation theory, Bronfenbrenner’s (1979) ecological systems theory, Bourdieu’s (2011) concept of social capital, and Schutz’s (1958) Fundamental Interpersonal Relations Orientation (FIRO) theory. Together, these frameworks provide a multi-level and motivational perspective: Berry highlights adaptation strategies (integration, separation, assimilation, marginalization); Bronfenbrenner emphasizes how nested systems—from classrooms to policy structures—shape adolescent development; Bourdieu explains how access to and conversion of capital depend on peer networks; and Schutz highlights three core interpersonal needs—inclusion, control, and affection—which illuminate the social motivations underlying NAS’s relational practices. Building on these foundations, the study also incorporates Social Exchange Theory and the principle of homophily to analyze cost–benefit dynamics of exchanges, the role of tie strength, and the influence of cultural similarity on peer selection.

A mixed-methods approach was employed, including a questionnaire administered to 51 NAS who have resided in Hong Kong for less than one year, and semi-structured interviews with 19 of them. The interviews explored social network composition, peer preferences, social activities, and perceived support. Findings indicate that NAS friendships fall into three categories: Mainland peers, Hong Kong peers with Mainland backgrounds, and local Hong Kong peers. These groups differ in cultural similarity and interaction frequency, offering complementary support functions—emotional support from culturally similar peers, and instrumental support from local peers. This classification illustrates how NAS’s peer ties operate as adaptive mechanisms, balancing cultural distance, relational costs, and diverse social needs within the combined framework of acculturation, ecology, social capital, and interpersonal motivation.

## 2. Literature Review and Theoretical Framework

### 2.1. Multidimensional Challenges and Resilience Mechanisms of Newly Arrived Students in Hong Kong

The term “Newly Arrived Students” (NAS) refers to students who have immigrated from Mainland China to Hong Kong within the past three years. This group faces distinct characteristics and challenges across cultural, educational, and psychological dimensions. One of the major difficulties lies in the formation of social networks, particularly in establishing peer relationships with local students and adjusting to Hong Kong’s cultural environment. These students encounter various educational challenges, such as language barriers (particularly in mastering Cantonese and English), difficulties adapting to new school systems, and academic setbacks, including grade repetition. W. H. Zhang and Lei (2009), in a longitudinal study on new immigrants in Shanghai, observed that adolescent migrants often experience “cultural shock” in transitioning between language systems and integrating into new educational frameworks. Their findings on academic delays mirror the grade duplication commonly seen among NAS in Hong Kong. Their study, using structural equation modelling, confirmed that peer groups with similar hukou (household registration) backgrounds can reduce adaptation stress by 43% (*p* < 0.001).

Cultural differences may also expose NAS to unfair treatment or exclusion from peers, which in turn affects their mental well-being and social integration. Studies have shown that NAS are not necessarily psychologically less resilient than their locally born counterparts; in fact, they may even outperform them on certain indicators of mental health. Firstly, NAS exhibit complex patterns in psychological adaptation. A comparative study by Lam et al. (2003) found that newly arrived students in Hong Kong scored higher on several mental health indicators than those born locally. However, this does not imply that NAS do not face challenges—particularly in language acquisition and school adjustment. Rao and Yuen (2001) noted that newcomers face significant difficulties in acquiring both English and Cantonese, which hampers their academic performance and social interactions. They highlighted that grade repetition may be necessary for some students, which not only delays academic progress but also affects their social development. Regarding social networks, Yuen (2010) argued that the identity construction of new arrivals is shaped by three main factors: place of birth, place of residence, and family influence. Critically, Bronfenbrenner’s (1979) ecological systems theory reveals these adaptation challenges as nested structures, explained that the developing person is embedded in a series of environmental systems ranging from immediate settings (microsystem) to overarching cultural patterns (macrosystem). For NAS, the school microsystem—where peer interactions occur—is constrained by exosystemic policies like Hong Kong’s language education framework that “create barriers to cross-group contact” (Johnson & Sandhu, 2007). Beyond language barriers, NAS also face social exclusion within the school microsystem. As Gao’s (2024) work demonstrates, the campus environment is often characterized by a monolithic Cantonese culture that can lead to de facto segregation, severely restricting meaningful academic and social engagement for newcomers from the Mainland. Phillion (2008) further emphasised the value of narrative research in understanding the lived educational experiences of these students. Research has shown that group counselling programmes can significantly enhance NAS’s self-esteem, particularly in areas related to peer relationships. NAS occupy a unique position within Hong Kong society, and their adaptation, social networks, and mental well-being have consistently drawn scholarly attention. These studies contribute to a deeper understanding of this population and suggest possible interventions to support their adaptation and development.

### 2.2. Cultural Adaptation and Adolescent Social Interaction

Adolescent social interaction is a core dimension of the cultural adaptation process, and scholars both in China and internationally have explored its underlying mechanisms from multiple perspectives. In contemporary cross-cultural psychology, cultural adaptation is often conceptualised through two dimensions: the maintenance of one’s heritage culture and the adoption of the host culture. Studies have demonstrated that cultural adaptation significantly influences the psychological well-being of immigrants, although findings on which specific strategies yield the best outcomes vary depending on context and methodology.

However, one major challenge highlighted in the literature is the cultural adaptation process within schools. For newly arrived students, successful adaptation involves not only meeting the cultural expectations of the school environment but also preserving connections to their home culture. Schools frequently exert “assimilation pressure” on ethnic minority adolescents, expecting them to conform to mainstream norms rather than supporting the development of a bicultural identity. As demonstrated by De Lise et al. (2024), the link between educational identity commitment and well-being appears to be especially salient for migrant youth. In sum, NAS’s psychological adjustment is closely linked to their experiences of cultural adaptation.

Moreover, the school environment plays a crucial role in shaping how adolescents adapt culturally, affecting their interactions both within school settings and the broader social context. For NAS, these adaptation processes are pivotal in determining whether they successfully integrate with local peers or remain confined to isolated networks composed of other new arrivals. Existing studies suggest that peer groups, online social practices, school ecology, and family dynamics are key factors influencing adolescent social interaction. Research by scholars has further localised and expanded theoretical understandings of these mechanisms. Firstly, peer groups play an irreplaceable and structurally significant role in adolescent socialisation. In a longitudinal study, Siennick and Osgood (2012) found that adolescents tend to choose peers with physical proximity and similar characteristics during unstructured social contexts, such as after-school hours. This preference for homophily is particularly pronounced among migrant youth. W. H. Zhang (2005), in a study on the social networks of Beijing’s migrant population, confirmed that adolescents with similar educational backgrounds or regional origins interact 38% more frequently than those from heterogeneous groups. Such homophilous connections effectively reduce communication costs during cultural adaptation. Becker and Luthar (2007) further indicated through quantitative research that prosocial behaviours, such as peer support, are central to gaining social preference. Bronfenbrenner’s (1979) ecological systems theory provides the final dimension of the CEMCCA framework, elucidating how environmental layers hierarchically regulate NAS’s acculturation strategies and capital conversion efficacy. Bronfenbrenner notes microsystem interactions are “the engine of development… but their power is moderated by institutional exosystems”. When schools lack “deliberate cultural brokerage programs” (Johnson & Sandhu, 2007), NAS compensate through pragmatic peer selection—using mainland-background peers as “microsystem interpreters”.

Friendship, as a key bond within peer groups, assumes unique functions in migration contexts. Hooijsma and Juvonen (2021) found that cross-group friendships significantly improve migrant adolescents’ attitudes towards the mainstream society. The positive effects of such friendships are moderated by cultural distance—when cross-group friends share partial cultural backgrounds, the attitude improvement was 29% higher compared to friendships with purely local peers. Jerrome (1984) emphasised the identity-building function of friendship as a foundational element of social networks. Alem et al. (2023) further illustrate that adolescents’ preference types—such as efficiency-loving or spiteful behaviors—are significantly correlated within friendship networks, highlighting the role of peer influence in shaping social attitudes during migration. Secondly, the proliferation of digital technology has reshaped the spatial-temporal dynamics of adolescent interaction, revealing a dual mechanism of influence in online socialisation. Van Zalk and Monks (2020) proposed a “rich-get-richer” effect, which manifests as socially capable students expanding their homophilous networks through platforms like WeChat, while socially anxious students rely on digital interaction to compensate for offline social deprivation. Colder Carras et al. (2017) found that although in-game interaction can alleviate adolescent social stress, excessive use may lead to a decline in real-life social skills.

Moreover, schools, as primary venues for adolescent socialisation, significantly shape interaction patterns through institutional design and peer group dynamics. Alem et al. (2023) developed the “peer preference typology theory,” which takes on a distinct form in Hong Kong schools: NAS tend to join “efficiency-oriented” peer groups focused on academic support, while “inequality-preferred” groups led by local students subtly exclude NAS. Ergin-Kocaturk et al. (2025) emphasize that social support, cultural capital, and psychological attributes play crucial roles in shaping international students’ adaptation to new cultural environments. Bourdieu further explains how power hierarchies constrain capital access, noting that the volume of social capital depends on the size of network connections one can effectively mobilize and the volume of capital (economic, cultural, symbolic) possessed by each connection (2011). This clarifies why NAS’ Cantonese deficiency—a cultural capital deficit—limits local tie mobilization despite high interaction frequency, forcing reliance on mainland-background peers as capital converters who “hold convertible resources across fields” (Zhou & Bankston, 1994).

Finally, the family, as the initial site of socialisation, may transmit risk factors that affect peer relationship formation. Sánchez-Sandoval and Verdugo (2021) found that adolescents experiencing financial stress or parent–child conflict at home are 2.7 times more likely to experience social isolation. The lack of familial support may lead NAS to rely more heavily on homophilous friendships to fulfil emotional needs. In this dynamic, parental attachment indirectly affects adolescents’ negative emotions through peer attachment, while also moderating the latter’s impact on emotional well-being.

### 2.3. Social Exchange Theory and Homophily

Social Exchange Theory (SET) posits that society is composed of continuous interactions among individuals, each aiming to maximise rewards while minimising costs. According to SET, social exchanges involve a series of interactions that generate mutual obligations. These exchanges are typically interdependent and contingent upon the actions of others. The essence of social exchange lies in human social needs—particularly how desires and emotions influence interpersonal communication and the exchange of social resources. Both social and economic exchanges are grounded in a fundamental characteristic of social life: many of the things we value and require—such as goods, services, and companionship—can only be obtained from others. People rely on one another for these valuable resources and engage in reciprocal exchanges to fulfil mutual needs. However, to comprehensively address NAS’s strategic balancing of cultural maintenance and host society engagement, this study integrates Berry’s (2005) acculturation theory. Berry defines acculturation as “the dual process of cultural and psychological change that takes place as a result of contact between two or more cultural groups”, emphasizing that individuals navigate adaptation through distinct strategies: integration (engaging both cultures), assimilation (adopting the host culture), separation (preserving heritage culture), or marginalization (rejecting both cultures).

This study introduces Schutz’s (1958) Fundamental Interpersonal Relations Orientation (FIRO) theory to examine the social needs of newly arrived students. The theory focuses on three core interpersonal needs in social interaction. The first is inclusion—the need to establish contact, engage with others, and maintain satisfying mutual relationships within a group. The second is control—the desire to exert influence over others or to be directed by others within a social hierarchy. The third is affection—the need to express and receive love or emotional closeness. Although similar to biological needs, this refers more specifically to the emotional bonds between individuals and their human environment. Schutz’s theory also considers behavioural compatibility in relationships, noting that individuals with similar needs—whether active or passive in expressing them—tend to be more compatible and can support each other in fulfilling unmet needs. In the context of NAS, these three social needs manifest in distinct ways. Inclusion is operationalized as the need to participate in classroom activities, group projects, and informal peer gatherings to avoid social isolation. Control relates to the desire to navigate the new school system effectively, seek academic help, and gain influence over one’s learning environment. Affection is defined as the need for emotional comfort, intimate self-disclosure, and a sense of belonging, which is crucial for psychological well-being. Brance et al. (2024) emphasize that social identity continuity and gain are pivotal pathways through which migrants navigate psychological challenges and foster resilience in new cultural contexts. This study examines how NAS strategically leverage different types of social ties to fulfil these multifaceted needs, often under constraints of cultural distance and linguistic barriers.

Beyond the traits of the relationships themselves, scholars have also turned their attention to the attributes of the actors involved. Burt (1995) argued that the value or influence of a tie depends on whether it bridges to a third party—a concept known as “structural holes.” McPherson et al. (2001) emphasised elements of homophily—such as ethnicity, gender, age, religion, education, occupation, and social class—which shape the likelihood and quality of interpersonal connections. McMillan (2019) found that adolescent immigrants tend to form friendships with peers who share similar migration backgrounds. He also observed that adolescents are more likely to bond with peers of the same ethnicity and gender. Building on this, Qiu and Qiao (2018) argued that in the job-seeking process, homophily among actors is more influential than the strength of their ties. The greater the similarity between actors, the higher the likelihood of receiving assistance through the connection, as shared traits foster mutual support. While Social Network Theory has traditionally been applied to employment-related interactions, some scholars have extended its application to examine the adaptation of immigrant populations. Liu (2019) suggested that non-kin neighbours provide social support to rural migrants in China, thereby aiding their integration into the host society. Zhao and Xu (2004) pointed out that strong connections with host community residents can reduce mutual distrust and facilitate social acceptance. Berry’s framework further clarifies this homophily preference, as cultural maintenance reduces communication costs during adaptation, yet over-reliance on separation may limit sociocultural integration. This tension aligns with our finding that NAS strategically deploy high-homophily weak ties for emotional needs while tolerating low-homophily strong ties for instrumental gains—a pragmatic integration strategy balancing costs and benefits.

However, while existing studies using social network theory have examined the adaptation of new arrivals in general, they have yet to fully address the experiences of younger members of this group. Current studies on NAS adaptation predominantly focus on structural factors such as ethnicity, gender, class, or cultural background, often overlooking the intersubjective dynamics within the NAS group itself. Even among research that does examine relational dynamics—i.e., intersubjectivity—the focus has largely been on the role of school-based friendships in adaptation, without differentiating the distinct types of friendships or their relative functions in the adaptation process. Finally, there remains a lack of research applying social network theory and homophily principles specifically to the adaptation of newly arrived youth in Hong Kong. To fill this gap, the present study employs the frameworks of social network theory and homophily to conduct both a survey and in-depth interviews with NAS in Hong Kong.

### 2.4. Cultural-Ecological Model (CEM)

To holistically explain how NAS navigate cultural dissonance, institutional barriers, and relational adaptation in Hong Kong, this study synthesizes Bronfenbrenner’s (1979) ecological systems theory, Berry’s (1990) acculturation framework, and Bourdieu’s (2011) capital typology into an integrated model—the Cultural-Ecological Model of Capital Conversion in Acculturation (CEM). Grounded in Bronfenbrenner’s ecological systems theory, this model conceptualizes NAS’s peer-network formation as a dialectical process shaped by multi-level environmental systems. Within this framework, Berry’s acculturation strategies and Bourdieu’s capital conversion operate dynamically across four hierarchical tiers.

Within Bronfenbrenner’s microsystem, shared Mandarin fluency, collective educational experiences and cultural familiarity minimize emotional costs and reinforce heritage identities. In immediate interaction settings (classrooms, dorms), NAS prioritize high-homophily relationships with Mainland peers to secure affective stability. These ties function as reservoirs of cultural capital (Bourdieu, 2011) that validate identity and minimize emotional transaction costs. In addition, social capital was found to moderate the association between immigration background and adolescent mental health (Delaruelle et al., 2021). As W. H. Zhang (2005) observed in migrant networks, such homophily reduces adaptation stress by 43%, offering a psychological buffer against cultural dislocation. This aligns with Berry’s separation strategy, where heritage-culture preservation provides emotional security amid host-society pressures.

The mesosystem—linking family and school—steers NAS toward pragmatic relationships with local peers. At the mesosystemic juncture, familial acculturation logics actively steer NAS toward instrumental bonds with local peers, as parents pragmatically encourage alliances for institutional capital acquisition—such as linguistic capital (Cantonese proficiency) and institutional capital (e.g., exam strategies), essential for academic survival (Sánchez-Sandoval & Verdugo, 2021). These instrumental ties exemplify Bourdieu’s institutional capital: local peers supply school-specific knowledge (e.g., exam systems) but demand high interaction costs due to cultural distance. As Baumert et al. (2024) found in a German context, cultural identification differentially predicts adjustment: host ties aid academic functioning, whereas heritage ties bolster psychological well-being.

Exosystemic policies actively constrain cross-group integration. As Berry established, immigrants typically adopt one of four strategies: integration (embracing both cultures), assimilation (adopting the host culture exclusively), separation (rejecting the host culture), or marginalization (disengaging from both). According to Suárez-Orozco and Suárez-Orozco (2024), schools must adopt an equitable, whole-child approach to effectively address their multifaceted challenges and foster an environment where they can truly thrive. The Hong Kong educational context, however, imposes institutionalized assimilation pressures (Johnson & Sandhu, 2007), manifesting as linguistic expectations and curricular norms. Without systemic support (e.g., bilingual curricula), NAS resort to homophilous consolidation, paradoxically limiting access to the very institutional capital they require for academic integration. This pressures NAS toward a pragmatic dual strategy, which often manifests as instrumental integration (cooperating with local peers for academic survival) alongside emotional separation (preserving emotional bonds with Mainland peers to sustain cultural identity).

At the macrosystem level, historical socio-political narratives and cultural hierarchies fundamentally configure peer dynamics. These macrosystemic asymmetries heighten dependence on mixed-background peers as cultural translators who reconcile heritage-host identities—a pragmatic integration strategy (Berry, 1990) that navigates cultural distance while preserving selfhood. Occupying Bourdieuian “structural holes” (Burt, 1995), these brokers convert capital across fields: explaining Cantonese idioms in Mandarin, decoding school bureaucracies, and legitimizing NAS’s hybrid identities. Their dual-cultural fluency embodies Berry’s integration strategy, resolving the assimilation-separation binary by bridging macrosystemic cultural distance through microsystemic trust. Thus, Macrosystemic cultural asymmetries elevate mixed-background peers as indispensable brokers.

**Figure 1:**
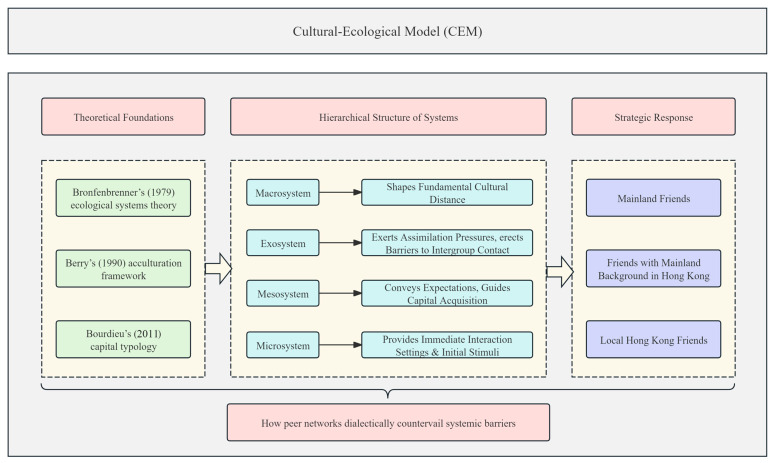
The Cultural-Ecological Model (CEM) of Social Integration Strategies among Newly Arrived Students (Bronfenbrenner’s (1979), Berry’s (1990) and Bourdieu’s (2011)).

The peer selection strategies of NAS extend beyond individual agency, emerging dialectically from nested ecosystemic forces: within the microsystem, high-homophily ties with Mainland peers secure emotional safety; at the mesosystem level, familial expectations propel instrumental bonds with local peers; across the exosystem, institutional policies erect barriers to cross-group contact; while in the macrosystem, cultural distance intensifies reliance on mixed-background peers as cultural brokers—a structural necessity for navigating divided social fields. Within this matrix, Berry’s acculturation manifests as relational tactics, Bourdieu’s capital as negotiable currency, and Bronfenbrenner’s ecology as the scaffold of constrained agency—collectively illustrating how peer networks dialectically countervail systemic barriers. To visually illustrate the integrated framework linking theoretical foundations, ecological system levels, and the differentiated strategic responses of NAS, we developed the Cultural-Ecological Model (CEM) (Figure 1).

## 3. Methods

### 3.1. Participants

This study focused on NAS in Hong Kong who had resided in the city for less than one year. The research was conducted at Scientia Secondary School, a popular institution among students from immigrant families. In 2024, NAS accounted for around 40% of the school’s total enrollment. With approximately 900 students enrolled, the estimated number of NAS reached 300, providing a sufficient sample pool for qualitative research.

A purposive sampling strategy was adopted to ensure the homogeneity of participants. All selected students met the criterion of having arrived in Hong Kong within the past 12 months. Although the Hong Kong government defines NAS as those who arrived within three years, the research team refined the scope to focus only on the most recent arrivals to better capture early-stage adaptation dynamics. Prior studies have highlighted that the first year of migration is particularly critical for social and psychological adjustment. For instance, He (2004) found in a longitudinal study of newly arrived women in Hong Kong that the first year constitutes a decisive period for social support construction, with online networks playing a buffering role in mitigating cultural shock and providing emotional resources. Similarly, L. Zhang and Shen (2025) demonstrated that Mainland immigrants often adopt avoidance or defensive strategies in their first year, gradually shifting towards collaborative and integrative identity negotiation in subsequent years. These findings collectively indicate that selecting participants who have resided in Hong Kong for less than one year allows this study to better capture the initial tensions and turning points of adaptation. Moreover, considering the limited number of interviews that could be carried out, the study further concentrated on students who had lived in Hong Kong for less than one year. This narrower focus allowed for a more precise understanding of their early adaptation experiences. Within this subgroup, random sampling was used to select participants. From this subgroup, 51 participants were randomly chosen for the initial survey. Subsequently, 19 students were selected for in-depth interviews based on a theoretical sampling approach. And Table 1 shows the sociodemographic characteristics of the interviewees. To justify the sample size, the study considered both theoretical saturation and research resource constraints. The number of 51 survey participants was sufficient to capture general patterns, while 19 in-depth interviews allowed the research team to reach data saturation, as no new themes emerged in the later stages of analysis. In terms of sampling, a purposive strategy was employed initially to ensure homogeneity, followed by random selection within the subgroup to reduce bias. To further guarantee coverage, at least two newly arrived students from each grade (except the final graduating year) were selected for interviews, thereby spanning a wide range of grade levels. Gender balance was also taken into account, with nine male and ten female interviewees. Nevertheless, purposive sampling may introduce limitations, as the participants were drawn from a single school, which could constrain the representativeness of the findings. It should be noted that the sample was drawn from a single school, which may limit representativeness. However, under the constraints of research resources, this sampling strategy allowed the study to specifically capture the social network characteristics of newly arrived students during their early stage of adaptation.

### 3.2. Research Design

A mixed-method design integrating structured questionnaires and semi-structured interviews was employed. The questionnaire served as the initial data collection tool to map basic information and social behaviours, while the interviews offered deeper insights into social network formation and peer interactions in the adaptation process.

The questionnaire served as an initial tool to gather baseline data and to inform the direction of subsequent interviews. It comprised four main sections: personal background information (such as arrival time and Cantonese proficiency), social network composition (including number of friends, peer preferences, and friendship criteria), social quality assessment (adapted from the Interpersonal Support Evaluation List to evaluate both objective and subjective aspects of social connection), and perceived social adaptation (focusing on school belonging, perceived support, and expectations for school support services). The questionnaire was distributed in paper format within classroom settings under the supervision of research assistants to ensure independence and completeness, with a 100% response rate. Based on the analysis of questionnaire responses, the research team developed a flexible interview guide.

The interviews were semi-structured, each lasting approximately 30–40 min. Mandarin was primarily used, supplemented with Cantonese or English when necessary. The researchers maintained a professional research relationship with participants to avoid classroom evaluation or hierarchical influence. The interview guide is provided in the Appendix A. The interviews were designed to delve deeper into the structure and function of students’ social networks, their criteria for selecting peers, the balance between online and offline social activities, and the emotional and instrumental functions of friendships. Emphasis was placed on understanding how different types of friends—such as Mainland peers, Mainland-background Hong Kong peers, and local Hong Kong students—contribute differently to the adaptation process. The interviews also encouraged students to reflect on their own perceived social effectiveness and articulate the forms of social support they felt they needed. Triangulation was applied by comparing questionnaire and interview data to validate findings and ensure internal consistency.

### 3.3. Procedure

A mixed-method design integrating structured questionnaires and semi-structured interviews was employed. The questionnaire served as the initial data collection tool to map basic information and social behaviours, while the interviews offered deeper insights into social network formation and peer interactions in the adaptation process. During the implementation of the study, the purpose, procedures, and potential risks were clearly explained to all participants, and written informed consent was obtained from both the students and their guardians. Recognizing that interviews might cause emotional distress for some students, the research team provided contact information for psychological support services so that assistance could be sought if needed. In addition, all data were anonymized during collection and storage, used solely for academic purposes, and handled under strict confidentiality measures to protect participants’ privacy. After receiving ethical approval from the school’s research committee and obtaining written informed consent from both students and their guardians, the researchers initiated the data collection in two stages.

First, 51 students participated in the questionnaire survey, which was conducted in a controlled environment to ensure independence and standardisation in responses. Following data collection, 47 valid responses were coded, digitised, and subjected to preliminary statistical analysis. The analysis results were then used to inform the selection of interview participants. 19 students were chosen using theoretical sampling, based on a combination of their sociodemographic characteristics and indicative patterns in their questionnaire responses. Before the interviews, written consent was again obtained from both students and guardians, with clear communication regarding the audio recording protocols. All interviews were conducted between 11 June and 27 June 2025, following the questionnaire phase. The interviews were conducted in a face-to-face format at the school in a quiet and private setting to encourage open communication. A semi-structured interview approach was adopted, which allowed the researchers to follow a prepared guide while also providing flexibility to explore new topics raised by participants. Mandarin was used as the primary language, with Cantonese or English supplemented when necessary, to ensure that students could express themselves comfortably. As interviews progressed, the research team adjusted the interview guide in real time to reflect emerging insights and to probe more deeply into relevant themes. This iterative process ensured that the data collected were not only rich and reliable but also closely aligned with the research objectives. To address potential emotional distress during interviews, the research team prepared counseling resources for participants. All data were anonymized and used solely for academic purposes, ensuring confidentiality and privacy. The entire data collection process adhered strictly to established ethical standards.

### 3.4. Data Analysis

Quantitative data from the questionnaires were processed using descriptive statistical methods to identify patterns in peer preferences, language ability, and perceived support. These results helped to frame the qualitative phase. Qualitative data from interviews were transcribed and thematically analysed using NVivo. The analysis followed a grounded theory approach, identifying codes, categories, and key themes through iterative reading. To enhance the reliability of the analysis, two researchers independently coded the data, and discrepancies were resolved through discussion until consensus was reached. Special attention was given to patterns of homophily, tie strength, and the allocation of emotional versus instrumental support within students’ peer networks. The combination of quantitative overview and qualitative depth enabled a comprehensive understanding of how newly arrived students construct and navigate their social worlds in Hong Kong.

### 3.5. Reliability and Validity Test

To evaluate the measurement properties, reliability and validity analyses were conducted. To assess the psychometric properties, we selected four items (C9: social satisfaction, D1: peer attitude, D2: sense of belonging, D3: integration and adaptation) to construct a measure of “social integration.” Internal consistency was assessed using Cronbach’s alpha, with the standardized alpha also reported to account for differences in item variances. Construct validity was examined through exploratory factor analysis (EFA). Prior to the analysis, Bartlett’s test of sphericity and the KMO index were employed to assess data suitability for factor analysis. Factors were extracted using the minimum residual method (minres), and model fit was evaluated using RMSEA, TLI, and RMSR indices.

## 4. Results

This section presents the test results of questionnaires and findings from the open and axial coding processes., focusing on the relationships and social satisfaction of NAS in Hong Kong. The results are structured around three main types of friendships: Mainland friends, local Hong Kong friends, and friends with Mainland background in Hong Kong. The analysis explores how each type of social support contributes differently to NAS’s social needs, based on the dimensions of tie strength and homophily.

### 4.1. Reliability and Validity Results

Reliability analysis indicated that the standardized Cronbach’s alpha was 0.81, exceeding the conventional threshold of 0.70, suggesting good internal consistency after controlling for variance differences. Bartlett’s test of sphericity was significant (χ^2^ = 21.39, df = 6, *p* = 0.002), indicating adequate inter-item correlations for factor analysis. EFA extracted a single factor explaining 30% of the variance. Factor loadings ranged from 0.39 to 0.72, with “peer attitude” (D1) and “sense of belonging” (D2) showing the strongest loadings (>0.60), while “social satisfaction” (C9) had the weakest loading (0.39). Model fit indices suggested acceptable unidimensionality (RMSEA = 0.052, TLI = 0.94, RMSR = 0.07), indicating that the four items reflected a single latent construct, albeit with modest explanatory power.

### 4.2. Open Coding

Through initial line-by-line coding of interview transcripts, we identified a series of concepts that reflect the lived experiences of NAS in Hong Kong. These open codes were then grouped into broader categories. Table 2 provides illustrative examples of this process.

The open coding stage highlights how everyday experiences—such as struggling with Cantonese in class, choosing friends based on shared hobbies, or maintaining emotional reliance on Mainland peers—were translated into conceptual categories like language adaptation, social motivation, or layered social networks. This process allowed us to capture both the practical and emotional dimensions of NAS’s adaptation.

### 4.3. Axial Coding

Building on the open coding, the axial coding process clustered these categories into seven core themes. These themes reflect the structural and cultural factors influencing NAS’s relational practices. Table 3 summarizes the core categories, their sub-categories, and representative concepts.

The axial coding results reveal that NAS’s social experiences are shaped both by structural factors (e.g., school language policies, class grouping, extracurricular activities) and cultural factors (e.g., linguistic distance, cultural identity, homophily in interests). Below we elaborate on each category.

### 4.4. Mainland Friends: Weak Ties—High Homophily

According to social tie theory, strong ties are characterized by frequent interaction, emotional closeness, and mutual dependency, while weak ties involve less frequent contact and lower emotional investment. Although NAS typically maintain weak ties with their Mainland friends due to geographical separation, these relationships remain significant due to high levels of homophily. Shared cultural backgrounds, language, educational experiences, and social norms reduce communication costs and enhance emotional understanding. According to the questionnaire, over 56% of NAS reported having fewer friends in Hong Kong than in the Mainland, while only 31.25% had more friends in Hong Kong.

Many students emphasized the emotional continuity provided by their Mainland friends, even though geographic separation limited daily interaction. These pre-existing friendships, formed before migrating to Hong Kong, offer a sense of cultural stability and identity reinforcement. For instance, FSS explained: “Whenever I need to vent, I always turn to my old friends from the Mainland… they are my only outlet for personal feelings.” This highlights how Mainland friends serve as safe spaces for affection needs despite weak tie strength.

Similarly, another student FXNX described the lack of belonging in Hong Kong by comparing her current friendships to “friends you meet on a tour group”, while emphasizing that only her Mainland friends provided deep emotional anchorage. These narratives resonate with Berry’s separation strategy, where students maintain strong cultural continuity through high-homophily ties. As one respondent, MYZM, remarked, “Hong Kong students have never experienced the Mainland high school entrance exam, so they can’t understand the pressure we went through.” This shared experience creates a foundation for emotional resonance and mutual understanding that newly formed relationships in Hong Kong cannot easily replicate.

Moreover, the familiarity with Mandarin, shared social media platforms (e.g., WeChat), and similar humor or values further facilitate meaningful communication and trust. Even though they are physically apart, NAS reported turning to their Mainland friends for emotional support, venting, or sharing secrets. As FLQJN expressed, “Whenever I have something funny or upsetting, I always turn to my friends from the Mainland.” These weak yet emotionally rich ties serve as a reliable source of affection support during the adaptation process.

In addition to emotional support, some students reported that Mainland friends acted as a symbolic link to their prior identity and educational history, especially in moments of stress or alienation. FHMR shared: “I didn’t adapt to the place, such as living habits, and then the unfamiliar language would make me a little uncomfortable. She (my old friend) would take the initiative to ask me how I was staying, and I would tell her about it.” While these friends could not provide physical companionship, their psychological presence offered emotional anchorage. Students also mentioned that they continued to share diaries or voice messages with Mainland friends, reinforcing mutual trust and continuity. From the coding perspective, these accounts align with the open codes of “emotional reliance on Mainland peers” and axial category of “Social Network Structure”. Mainland friends thus function as emotional anchors and identity stabilizers, reducing their adaptation stress.

### 4.5. Local Hong Kong Friends: Strong Ties—Low Homophily

In contrast to their ties with Mainland friends, NAS develop strong ties with local Hong Kong classmates through frequent face-to-face interactions in school. These relationships play a practical and instrumental role in fulfilling inclusion needs—such as help with schoolwork, guidance on school systems, and daily companionship. However, due to low homophily in language, social background, and cultural experience, these friendships often lack deeper emotional connection. While 89.58% of students reported having at least one Hong Kong friend, none indicated a preference for befriending local students. 75% claimed to have no clear preference.

Although most NAS initially worried about potential prejudice from local peers, many reported positive experiences. Students repeatedly mentioned how local peers provided help with academic tasks and daily school routines. For example, FLJQN noted: “When they saw that I couldn’t understand Cantonese, they would come over to help me… They even explained the meaning of Cantonese phrases I didn’t know.” MCZF also described how a local classmate helped translate during lessons when he struggled to understand the teacher. However, language barriers continued to limit deeper interaction and these ties were seldom used for emotional disclosure. FMNF said, “They speak Cantonese too fast—I can’t join the conversation.” “At school I only have one or two I can talk to. I hope to have closer friends here, but there is always a gap in topics with local students.” Additionally, differences in social media usage (e.g., WhatsApp and Instagram vs. WeChat) and cultural references further reduced shared interests and communication comfort.

Furthermore, students expressed strategic considerations in maintaining these relationships. For example, FLQJN mentioned that although she attempted to switch to using WhatsApp like her local classmates, she found it “too troublesome” and ultimately chose to retain her habits. Similarly, FLJX considered learning Cantonese to better integrate but chose instead to focus on core academic subjects due to time constraints. These decisions reflect a rational evaluation of social cost versus expected benefit, aligning with Social Exchange Theory. Interviews also revealed that NAS seldom expected long-term friendship development with local peers. MCZF explained: “I want to be friends with people who share similar values, but I don’t think that’s possible with most local Hong Kong students.”

As a result, NAS often described their local Hong Kong friends as “school-only” companions. FWYR stated she only socializes with local peers during class time, with minimal interaction outside school. Emotional self-disclosure was rare in these relationships, as FMYF noted: “If you talk about negative emotions, you can only do it jokingly. You can’t be too honest—it makes the other person uncomfortable too.” The high cost of emotional exposure, coupled with low cultural understanding from local peers, discourages NAS from investing in these friendships beyond academic or instrumental support. This pattern corresponds to the axial category of “Social Needs and Emotional Value”, showing that local ties primarily satisfy inclusion and control needs, but not affection. From a theoretical perspective, these relationships reflect that NAS weigh the high cost of cross-cultural communication against practical returns such as translation, exam strategies, and companionship.

### 4.6. Friends with Mainland Background in Hong Kong: Strong Ties—High Homophily

Among all peer types, Hong Kong students with Mainland backgrounds emerged as the most balanced type of support. These peers share both the proximity of daily school interaction (strong ties) and the cultural familiarity of similar upbringing and migration background (high homophily). 25% of students indicated a preference for making friends with Mainland peers, while 0% preferred local students. As a result, NAS reported receiving the broadest range of social support—including companionship, academic assistance, and emotional understanding—from this group.

NAS often relied on these friends for academic adaptation. Several interviewees mentioned receiving help with language learning, especially Cantonese, or with understanding the local curriculum. FHMR explained, “One of my friends had been in a DSE-focused school, so I asked her for help in English and science.” These friends’ dual familiarity with both Mainland and Hong Kong systems made them valuable cultural and academic mediators. In several cases, these peers also acted as cultural translators, not only helping NAS understand classroom instructions but also explaining social norms, slang, or even cafeteria routines. FMYF described one such friend as someone who “had been in Hong Kong since primary school, so she understood both sides. When I didn’t understand something, she would explain it in Mandarin and help me get used to things.” Such relationships reduced the cognitive load of adaptation and increased NAS’s sense of belonging.

Moreover, these relationships frequently began in classroom settings or through mutual Mainland acquaintances and quickly developed into close relationships. As FMYF described, “We’re like ‘dazi’ (buddies) at school—eating and doing everything together.” These relationships fulfilled not only shallow social needs (e.g., daily interaction) but also deeper needs for trust, empathy, and emotional sharing. Students highlighted that communicating with these peers required less effort, involved less risk, and yielded greater emotional and practical returns.

Additionally, students expressed a desire to deepen these connections long term. MHXY stated: “Although I share some values with my local classmates, it’s hard to find someone with the same interests. With Mainland-background friends, we understand each other better and I want to stay close to them.” Another student FWYR described this group as reliable companions who bridged emotional and instrumental needs: “If it’s a school-related problem, I talk to my Hong Kong friends, and I think after a period of interaction, one of them has chance to be my best friend. Maybe at that time, I would like to share more personal matters with her.” These friends were not only bridges, but anchors for sustained social development. Over time, these relationships became vital for NAS’s adjustment. The high levels of both trust and shared experience fostered a strong sense of belonging and reduced feelings of isolation. As MHXY stated, although he shared values with local students, he still found it difficult to connect due to differing interests. Thus, he preferred to deepen ties with peers who had a Mainland background.

This dual role reflects both Berry’s integration strategy and Bourdieu’s idea of capital conversion: Hong Kong peers with Mainland background help newcomers translate cultural codes and access institutional capital without sacrificing emotional resonance. In the coding structure, these accounts correspond to the axial category of “Support Systems and Expectations” and “Social Network Structure”, confirming their role as bridges between Mainland and Hong Kong systems.

**Figure 2:**
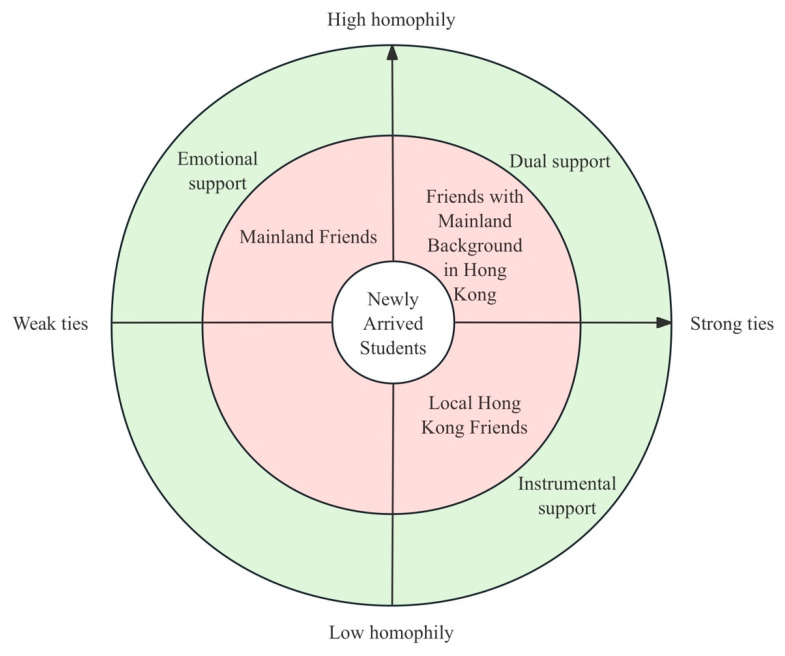
Social Relationship Matrix of Newly Arrived Students.

To sum up, the results demonstrate that NAS’s adaptation is not a linear process of “integration” but a dynamic negotiation shaped by both structural constraints (educational policies, institutional contexts) and cultural factors (linguistic barriers, identity, homophily). According to Figure 2, their friendships form a layered architecture: emotional anchoring from Mainland friends, instrumental support from local Hong Kong friends, and dual emotional-instrumental support from Hong Kong friends with Mainland background. This social portfolio reveals a pragmatic balancing of costs and benefits, consistent with Social Exchange Theory and the Cultural-Ecological Model of Capital Conversion in Acculturation.

This figure illustrates the relationship between tie strength and homophily across three types of friendships observed among NAS. It also maps out the corresponding forms of social support provided: emotional support, instrumental support, or dual support.

## 5. Discussion

This study illuminates the complex social strategies employed by NAS in Hong Kong as they navigate the challenges of cultural adaptation, linguistic barriers, and academic integration. Grounded in the Cultural-Ecological Model of Capital Conversion in Acculturation (CEM), the findings reveal that NAS actively construct a multi-layered social network to fulfil distinct interpersonal needs through differentiated types of friendships. These relational strategies are not merely reactive but reflect a dynamic negotiation between cultural homophily, tie strength, and institutional constraints—each layer corresponding to a specific ecosystemic level within the CEM framework.

At the microsystem level, NAS maintain weak ties with Mainland peers, characterised by high cultural homophily and shared Mandarin linguistic capital. These relationships function as reservoirs of emotional support and cultural continuity, effectively reducing the psychological cost of adaptation. This aligns with Berry’s separation strategy and Bourdieu’s concept of cultural capital, whereby familiar symbols and shared experiences serve as buffers against cultural dislocation. As reported by respondents, these ties provide affective stability and identity reinforcement, fulfilling Schutz’s need for affection despite geographical separation.

Conversely, strong ties with local Hong Kong peers—situated within the mesosystem—are primarily instrumental. Facilitated by daily school interactions, these relationships help NAS acquire institutional capital, such as Cantonese language skills and academic knowledge, essential for navigating the new educational environment. However, low cultural homophily and limited emotional reciprocity constrain their depth, reflecting the high transaction costs associated with cross-cultural engagement. This pragmatic orientation resonates with Social Exchange Theory, as NAS weigh relational investments against practical returns, often prioritising academic survival over emotional intimacy.

Most significantly, friendships with Hong Kong peers of Mainland background occupy a unique brokerage position across micro-, meso-, and macrosystems. These relationships combine high homophily with strong ties, enabling dual emotional and instrumental support. These peers act as cultural translators and institutional guides, bridging structural holes between heritage and host cultures. This role exemplifies Berry’s integration strategy and Bourdieu’s notion of capital conversion, wherein individuals leverage dual cultural fluency to access resources across social fields. Within Bronfenbrenner’s ecology, these friends help mitigate exosystemic barriers—such as monolingual school policies—by facilitating microsystemic integration.

**Figure 3:**
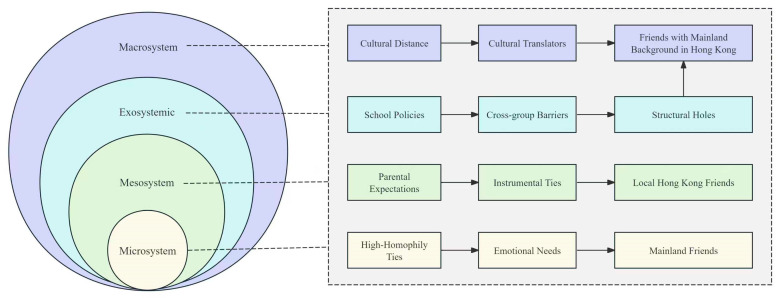
Layered Functions of Friendship in NAS’s Social Network.

To visualize this layered support system, Figure 3 below illustrates how NAS situate different friendship types within concentric social circles. Each layer corresponds to a particular function, from emotional anchoring to academic support to broader cultural integration.

This figure maps the positioning of three friendship types relative to the core group of NAS, highlighting their roles in navigating contextual challenges and facilitating adaptation.

As shown in the figure, Mainland friends form the innermost emotional ring, providing cultural continuity in times of stress. Friends with similar backgrounds in Hong Kong constitute the middle layer, facilitating both emotional bonding and practical help. Local Hong Kong friends occupy the outer ring, representing exposure to the host culture and facilitating school-based integration. However, this outer ring also represents the greatest distance in shared identity, which may explain the limited emotional returns. This stratified social structure reflects a pragmatic adaptation strategy. Rather than pursuing one “ideal” type of friend, NAS selectively engage with different peers based on expected costs and benefits. This aligns with Social Exchange Theory, which suggests that individuals invest in relationships proportionate to the perceived return on emotional and informational resources. Furthermore, from a developmental perspective, this layered network may not be static. Over time, some weak ties may strengthen and facilitate cross-layer movement, especially as NAS improve their language proficiency and gain confidence in intercultural contexts. Future studies may explore how mobility within these social circles influences long-term integration outcomes.

The findings suggest that enhancing NAS’s adaptation requires coordinated efforts across multiple ecosystemic levels. The Hong Kong government and cross-departmental mainland-Hong Kong committees should formulate integration policies that recognize the complementary functions of different social ties. This includes funding structured bilingual and cultural bridge programs, supporting after-school language acquisition initiatives, and promoting inclusive educational policies that reduce institutional assimilation pressures. Schools play a critical mesosystemic role. They should implement peer-mentoring systems that pair NAS with both mainland-background and local students, thereby legitimizing dual support strategies. Teacher training on multicultural sensitivity, the integration of NAS perspectives into school activities, and the creation of culturally mixed task teams can further facilitate meaningful interaction and diminish segregation. Families of NAS can be empowered through parental guidance sessions and cross-cultural communication workshops to better understand the school system and emotional needs of their children. Encouraging parental involvement in school activities and fostering an open attitude toward both heritage and host cultures can strengthen emotional security and facilitate smoother integration.

NAS themselves may benefit from guided self-reflection on their relational strategies, opportunities to develop cross-cultural communication skills, and encouragement to engage cautiously yet openly with local peers. Student-led cultural exchange activities and psychosocial support clubs can also provide safe spaces for identity negotiation and emotional expression. Finally, mainland and Hong Kong educational authorities could collaborate to establish portable credential systems, shared teacher training programs, and student exchange initiatives that normalize population mobility and reduce structural friction in academic integration.

In summary, social network is not merely a byproduct of adaptation but a mechanism through which NAS actively manage their social, emotional, and academic transitions. The stratified structure of NAS’s social networks underscores a strategic response to ecosystemic pressures. Rather than adopting a singular acculturation strategy, NAS deploy a relational portfolio that balances emotional security with practical necessity. This nuanced approach challenges binary narratives of assimilation versus separation, highlighting instead a context-sensitive and dynamic process of negotiation. The differentiated functions of three friendship types—emotional grounding from Mainland friends, dual support from Mainland-background peers, and instrumental assistance from local peers—together form a composite network architecture that reflects both resilience and strategy.

## 6. Conclusions

This study explored the dynamic adjustment mechanisms in satisfying the social needs of Newly Arrived Students (NAS) in Hong Kong, with a focus on how different types of peer relationships—Mainland friends, friends with Mainland background in Hong Kong, and local Hong Kong friends—differ in their capacity to offer emotional or instrumental support. By integrating Berry’s acculturation theory, Bronfenbrenner’s ecological systems theory, Bourdieu’s concept of social capital, and Schutz’s FIRO theory, the study highlights that NAS’s peer strategies are systematically shaped by cultural distance, ecological constraints, resource availability, and interpersonal motivations. This provides a comprehensive explanation of how emotional and instrumental needs are balanced through layered networks.

Instead of treating friendships as homogeneous, the findings underscore that NAS maintain a portfolio of ties: some offering emotional security, others providing institutional resources, and still others bridging cultural gaps. This portfolio approach demonstrates that adaptation is not linear but involves pragmatic negotiation across contexts. Rather than imposing a one-size-fits-all integration model, schools and policymakers should design multi-layered support: fostering emotional stability through peer mentoring, reducing institutional barriers via bilingual programs, and legitimizing cultural brokerage roles. Such systemic efforts can enhance both emotional resilience and institutional adaptation among NAS.

This study contributes theoretically by linking micro-level peer choices with macro-level institutional structures, offering a more holistic picture of youth migration adaptation. Future research could employ longitudinal or comparative designs to examine how these layered strategies shift over time, and how different school ecologies reinforce or hinder adaptive mechanisms. Such work would extend understanding of migrant youth resilience beyond Hong Kong, contributing to global debates on migration and education.

Through the lens of the Cultural-Ecological Model (CEM), the study further elucidates the dynamic and strategic nature of social network formation among NAS in Hong Kong. It reveals that their peer relationships are not formed in a vacuum but are shaped by multi-layered ecosystemic forces—from micro-level emotional security sought through high-homophily ties, to meso-level instrumental gains from local connections, all the way to macrosystemic cultural and institutional constraints. These relationships collectively form an adaptive structure that enables NAS to navigate cultural distance and fulfil diverse social needs. The findings underscore the importance of moving beyond simplistic integration-assimilation binaries and instead recognizing the agency and strategy inherent in migrants’ social practices. Future policies and school-based interventions should therefore adopt an ecological perspective, fostering environments that acknowledge and support the complementary functions of these varied social ties in facilitating sustainable adaptation.

This study inevitably has several limitations. The sample was drawn from a single secondary school, limiting representativeness, and the relatively small cohort narrowed the diversity of perspectives. Data were based on self-reports from minors, which may have been influenced by recall bias or social desirability, despite ethical safeguards. Methodologically, the study remained exploratory, employing descriptive statistics and grounded theory coding without advanced quantitative models or longitudinal tracking, which restricts causal claims. Future research should build on this work with theory-driven hypotheses, multi-site sampling, and follow-up designs to capture the evolving nature of adaptation. Despite these constraints, the findings provide novel insights into the interplay between cultural adaptation, social capital, ecological constraints, and interpersonal needs, offering both theoretical advancement and practical guidance for supporting migrant youth.

## Figures and Tables

**Table 1:** Sociodemographic Characteristics of Interviewees.

Interviewee ID	Gender	Age	Grade
FCPJ	Female	12	S1
FLQJN	Female	12	S1
FSS	Female	14	S2
MHXY	Male	15	S4
FWYR	Female	17	S5
MLZM	Male	13	S1
MTLY	Male	12	S1
FHMR	Female	13	S2
MCZF	Male	16	S4
FLJX	Female	18	S4
FYXT	Female	12	S1
FYLY	Female	15	S1
MDJ	Male	14	S2
MYZM	Male	16	S3
FMYF	Female	18	S5
MLJX	Male	14	S1
FXNX	Female	12	S1
MHSH	Male	14	S2
MBYB	Male	14	S3

Note: The coding is based on a combination of the respondent’s gender and initials of their names.

**Table 2:** Example of Open Coding.

Interview Excerpt	Conceptualisation	Category
“I couldn’t understand the Cantonese in class, so I asked a classmate to translate.”	Cantonese barrier; reliance on peer translation	Language adaptation
“I like to make friends with classmates who also enjoy drawing.”	Interest-driven friendship	Social motivation
“In Hong Kong I usually hang out with my classmates, but I still share my secrets with my old friends from the Mainland.”	Emotional reliance on Mainland peers; daily companionship from local peers	Layered social networks
“During music class line-up, a classmate came to me and said ‘let’s be friends’, that’s how I got to know her.”	Passive acceptance of friendship; initiated by others	Social mode
“I’m afraid of being left out, so I try to approach others proactively.”	Fear of isolation; proactive expansion	Social belonging
“I hope the school can provide us with more opportunities to socialise.”	Expectation of institutional support	Support system needs

**Table 3:** Axial Coding Table.

Core Category	Sub-Categories	Concepts
Language and Learning Adaptation	Cantonese barriers, Mandarin as bridge, English learning pressure	Linguistic distance; cross-linguistic communication; classroom adaptation
Social Mode	Interest-driven, passive acceptance, proactive expansion	Homophily of interests; differentiated interaction modes
Social Network Structure	Mainland weak ties (emotional), Local strong ties (instrumental), Mixed-background strong ties (dual support)	Layered social structure; complementary social functions
Social Arenas and Triggers	Classroom grouping, extracurricular clubs, online chat groups	Informal interaction settings; institutionalised opportunities
Social Needs	Humorous peers, meal partners, avoiding isolation	Emotional support; belonging needs
Social Belonging	Tourist mentality, reliance on Mainland ties, gradual integration, dual identity	Cultural identity construction; differentiated sense of belonging
Support Systems	Cantonese courses, social skills training, cross-class activities	Institutional support; cross-cultural bridging

## Data Availability

The data supporting this study’s findings are not publicly available due to privacy and ethical restrictions.

## Appendix A. The Guideline of Interview

**Study Title:** Cultural Distance and Social Needs: The Dynamic Adjustment Mechanisms of Social Support Among Newly Arrived Students in Hong Kong
**Interview Topic:** Social Networks and Social Needs of Newly Arrived Secondary Students in Hong Kong
**IRB Approval No.:** HIT-2025043

**Interviewer Introduction & Protocol:**

The semi-structured interviews were conducted by members of the core research team, all of whom have received formal training in qualitative research methods and interview techniques.

I. *Primary Interviewer:* Shiyi Zhang

Affiliation: Department of Sociology, The Chinese University of Hong Kong, Hong Kong S.A.R.; Department of Sociology, Harbin Institute of Technology, Harbin, China.

Role: Zhang designed the interview guide, conducted the majority of the interviews, and led the qualitative data analysis. Her/his dual background in Mainland China and Hong Kong provided valuable cultural and linguistic insights for engaging with the participant pool.

II. *Co-Interviewer:* Qi Wu

Affiliation: Department of Sociology, Harbin Institute of Technology, Harbin, China.

Role: Wu assisted in coordinating the interview logistics and contributed to the preliminary coding and analysis of the transcripts.

III. *Principal Investigator (PI):* Xuhua Chen

Affiliation: Department of Sociology, Harbin Institute of Technology, Harbin, China.

Role: Prof. Chen provided overall supervision for the research project, including the formulation of the research design, methodological guidance, and ethical oversight.

All interviews were conducted in Mandarin Chinese, the native language of both the interviewers and the participants, to ensure clarity and comfort during conversations. Prior to each interview, the study’s purpose was reiterated, informed consent was verbally confirmed, and permission for audio recording was obtained. The interviews lasted between 45 and 60 min and were conducted in a private and quiet setting to ensure confidentiality. The following guide outlines the core themes and questions used to structure the discussions; interviewers employed prompts and follow-up questions as needed to explore relevant topics in depth.

**Interview Protocol & Core Questions:**

I. *Social Preferences and Social Network Formation*

1. What kind of people do you generally prefer to make friends with? Are you more inclined to befriend those who share commonalities with you, or those whom you admire or look up to?

2. What characteristics do you typically value in others when forming friendships? How do factors like personality, appearance, capabilities, academic performance, or hobbies influence your willingness to befriend someone? Could you elaborate with specific examples?

3. How did you meet and develop a friendship with your current best friend? What are they like? Why do you think you became close friends?

4. Since coming to study in Hong Kong, have you made new friends? If so, could you share how you met and got to know them?

5. In your current social circle, do you have more friends you met after arriving in Hong Kong, or more old friends from the past?

6. Do you perceive different traits between friends from Hong Kong and those from the Mainland? Are there differences in how you communicate with them or the topics you discuss?

II. *Social Needs and Social Evaluation*

1. What activities do you usually do with your friends? (e.g., what do you play, talk about?) Is online interaction more common than face-to-face interaction? Could you share any particularly memorable shared experiences you’ve had with friends so far?

2. How frequently do you participate in classroom interactions and extracurricular activities at school? How do you feel during these participations? During breaks, lunchtime, or after school, do you usually spend time alone or with friends?

3. Were there any aspects you found difficult to adapt to when you first started studying in a Hong Kong secondary school? (e.g., academics, interpersonal relationships, culture, environment…). Compared to your previous school life in the Mainland, what differences do you perceive?

4. During your adaptation process, have friends provided any help? If yes, what specific help did newly made friends versus old friends offer? Please provide examples. If not, do you feel the absence of friends has impacted your adaptation and integration into the learning and life here?

5. Since arriving in Hong Kong, have you ever felt anxious about making friends? Have you experienced loneliness? Are there specific moments when you felt a strong need for friends’ companionship?

6. If you haven’t made new friends since arriving, have you attempted to make connections or initiate communication? If so, what difficulties did you encounter in this process? (e.g., language barriers, cultural differences, limited time together…?)

7. Overall, are you satisfied with the current state of your social circle (number of friends, quality of interactions)? If satisfied, could you explain why? If not satisfied, what kind of help do you feel you need?
